# FTO-targeted siRNA delivery by MSC-derived exosomes synergistically alleviates dopaminergic neuronal death in Parkinson's disease via m6A-dependent regulation of ATM mRNA

**DOI:** 10.1186/s12967-023-04461-4

**Published:** 2023-09-22

**Authors:** Yan Geng, Xinyi Long, Yuting Zhang, Yupeng Wang, Guoxing You, Wenjie Guo, Gaoming Zhuang, Yuanyuan Zhang, Xiao Cheng, Zhengqiang Yuan, Jie Zan

**Affiliations:** 1 School of Biomedical and Pharmaceutical Sciences, Guangdong University of Technology, Guangzhou, 510006 China; 2 Department of Radiology, Guangzhou Panyu Central Hospital, Guangzhou, 511400 China; 3 The affiliated TCM Hospital of Guangzhou Medical University, Guangzhou, 510130 China; 4 State Key Laboratory of Dampness, Syndrome of Chinese Medicine, The Second Affiliated Hospital of Guangzhou University of Chinese Medicine, Guangzhou, Guangdong China; 5 Provincial Key Laboratory of Research on Emergency in TCM, Guangzhou, China; 6 Department of Neurology, Guangdong Provincial Hospital of Traditional Chinese Medicine, Guangzhou, 510120 China

**Keywords:** Parkinson’s disease, N6-methyladenosine modification, FTO, siRNA, Exosomes

## Abstract

**Background:**

Parkinson's disease (PD), characterized by the progressive loss of dopaminergic neurons in the substantia nigra and striatum of brain, seriously threatens human health, and is still lack of effective treatment. Dysregulation of N6-methyladenosine (m6A) modification has been implicated in PD pathogenesis. However, how m6A modification regulates dopaminergic neuronal death in PD remains elusive. Mesenchymal stem cell-derived exosomes (MSC-Exo) have been shown to be effective for treating central nervous disorders. We thus propose that the m6A demethylase FTO-targeted siRNAs (si-FTO) may be encapsulated in MSC-Exo (Exo-siFTO) as a synergistic therapy against dopaminergic neuronal death in PD.

**Methods:**

In this study, the effect of m6A demethylase FTO on dopaminergic neuronal death was evaluated both in vivo and in vitro using a MPTP-treated mice model and a MPP + -induced MN9D cellular model, respectively. The mechanism through which FTO influences dopaminergic neuronal death in PD was investigated with qRT-PCR, western blot, immumohistochemical staining, immunofluorescent staining and flow cytometry. The therapeutic roles of MSC-Exo containing si-FTO were examined in PD models in vivo and in vitro.

**Results:**

The total m6A level was significantly decreased and FTO expression was increased in PD models in vivo and in vitro. FTO was found to promote the expression of cellular death-related factor ataxia telangiectasia mutated (ATM) via m6A-dependent stabilization of ATM mRNA in dopaminergic neurons. Knockdown of FTO by si-FTO concomitantly suppressed upregulation of α-Synuclein (α-Syn) and downregulation of tyrosine hydroxylase (TH), and alleviated neuronal death in PD models. Moreover, MSC-Exo were utilized to successfully deliver si-FTO to the striatum of animal brain, resulting in the significant suppression of α-Syn expression and dopaminergic neuronal death, and recovery of TH expression in the brain of PD mice.

**Conclusions:**

MSC-Exo delivery of si-FTO synergistically alleviates dopaminergic neuronal death in PD via m6A-dependent regulation of ATM mRNA.

**Supplementary Information:**

The online version contains supplementary material available at 10.1186/s12967-023-04461-4.

## Introduction

Parkinson's disease (PD) is the second neurodegenerative disease, which mainly occurs in older people [1]. The common pathological manifestations of PD are the gradual loss of dopaminergic neurons in the substantia nigra and striatum, and abnormal accumulation of a-synuclein (α-Syn) [2]. Until now, clinical use of dopamine replacement for PD therapy could only alleviate symptoms, but could not prevent the PD progression [3]. Thus, it is essential to fully elucidate the pathological mechanism of dopaminergic neuron death and develop more effective therapies for PD.

As the most prevalent modification of mRNAs in eukaryotic cells, the N^6^-methyladenosine (m6A) modification regulates mRNA splicing, stability and nuclear export, and plays essential roles in various cellular processes. The m6A level is modulated by methyltransferases and de-methyltransferases [4]. m6A modification is highly abundant within the brain and has been found to be involved in nervous system development and its dysregulation is closely associated with neurodegenerative diseases [5, 6]. For example, accumulation of methyltransferase-like 3 (Mettl3) is identified in the insoluble fractions of the postmortem brain samples of patients with Alzheimer's disease, which is positively correlated with the examined levels of insoluble Tau protein [7]. The reduction of m6A level in the striatum region of brain results in significant decrease of the neurotransmitter dopamine [8]. Twelve m6A-associated single-nucleotide polymorphisms have been revealed to be significantly associated with the PD risk [9]. These studies indicate that m6A modification plays important roles in the PD development. Therefore, it is essential to elucidate the underlying mechanisms for the modulation of dopaminergic neuronal death by m6A modification for developing new therapeutic drugs.

Nowadays, sufficient blood–brain barrier (BBB) crossing for drug delivery to central nervous system is still a key challenge in neurological therapeutics [10]. Recently, more and more attention has been focused on mesenchymal stem cells (MSCs), which is one of the very commonly investigated adult stem cell types with multipotential differentiation and self-renewal capacity and could cross the BBB [11, 12]. MSCs have been reported to present great therapeutic potential for many diseases, including COVID-19, neurodegenerative diseases, cardiovascular disease, acute kidney injury, cancer and so on [13–15]. Interestingly, increasing evidences showed MSCs-derived exosomes (MSC-exo), a subtype of extracellular vesicles with approximately 40–150 nm size and containing various biologically active molecules like proteins, lipids and RNAs, are mostly responsible for the therapeutic effects of MSCs [16]. Due to their innate tropism towards injured tissue, low immunogenicity, anti-inflammation, and high clinical safety, MSC-exo have been employed as a tissue damage-targeted drug delivery vehicle [17]. Our previous studies revealed that MSC-exo could be engineered to co-deliver the proapoptotic factor TRAIL and some chemo-drugs or CDK9-targeted siRNAs as targeted combination therapies against lung cancer, hepatocarcinoma and neuroblastoma [18, 19]. A recent study reports that exosomes from hypoxic MSCs promote cerebral angiogenesis, brain remodeling and neurological recovery after focal cerebral ischemia in mice [20]. MicroRNA-22-encapsulted MSC-exo have been shown to attenuate neuroinflammation and improve neurological function in the murine model of Alzheimer's disease [21]. Therefore, MSC-exo could also be potentially utilized to deliver therapeutic agents for PD therapy as well.

In this study, we aim to investigate how m6A modification regulates dopaminergic neuronal death in PD and its underlying mechanism in cellular and animal models of PD, and explore to harness MSC-exos to deliver therapeutic siRNAs as a novel m6A modification-targeted PD therapy.

## Methods

### Cell culture

Mouse dopaminergic neuronal MN9D cells were cultured in high glucose-containing DMEM supplemented with 10% fetal bovine serum in 95% humidified air and 5% CO_2_ at 37 ℃. Human umbilical cord-derived MSCs were cultured in DMEM/F12, as our previously described [19].

### Isolation of exosomes

Exosomes were isolated from the cell culture medium of MSCs, as our previously described [18].

### Characterization of isolated exosomes

Isolated exosomes were detected using the transmission electron microscopy (TEM) to observe the morphology of exosomes, and using a nanoparticle flow cytometer (nanoFCM, Xiamen, China) to determine the size distribution of exosomes, as our previously described [18].

### Mice PD model

Male C57BL/6 mice (26–30 g, 4-month-old) were obtained from the Animal Research Centre of Guangzhou University of Chinese Medicine (Guangzhou, China) and were housed in a room kept at 24 ± 2 °C temperature and approximately 40% humidity in a 12 h dark/12 h light cycle. Before being divided into experimental groups, all animals had free access to standard food and water. The study protocol is approved by the Institutional Animal Care and Use Committee of Guangdong University of Technology (No.20210721), and all animal experiments were carried out in accordance with the the National Institutes of Health guide for the care and use of Laboratory animals. Male C57BL/6 mice were intraperitoneally injected with MPTP hydrochloride (30 mg/kg/day, Sigma, USA) for 5 consecutive days. Control mice were injected with equal volume of 0.9% saline. 3 days after the last injection of MPTP, behavioral tests were performed.

### Morris water maze test

Three days after MPTP injection, each group of mice was put into a round pool for the Morris water maze test, as previously described [22].

### Cell viability assay

Cell viability was detected with the CCK-8 Cell Counting Kit (Vazyme, China), according to the manufacture’s instruction. Briefly, MN9D dopaminergic neurons were treated with different concentrations of 1-methyl-4-phenylpyridinium (MPP +) (0, 100, 200, 500, 1000 μM) for 24 h. Then, 10 μI of CCK-8 solution was added to each well for 1 h. Finally, the absorbance at 450 nm was measured.

### Immunofluorescence

Immunofluorescence was performed as our previously described [23]. The primary antibodies, including alpha synuclein antibody (α-syn, 10842-1-AP, Proteintech, China), TH antibody (25859-1-AP, Proteintech, China), FTO antibody (27226-1-AP, Proteintech, China), and ATM antibody (27156-1-AP, Proteintech, China) were used.

### Immuno-Histochemistry (IHC) analyses

Three days after the last injection of MPTP, mice were sacrificed and brain tissues were resected for IHC analyses, as our previously described [15]. The brain tissues were stained with m6A antibody (68055-1-Ig, Proteintech, China), α-syn (10842-1-AP, Proteintech, China), FTO antibody (27226-1-AP, Proteintech, China), and the TUNEL assay kit (C1090, Beyotime, China), respectively.

### Determination of the m6A level

The m6A level in total RNA was detected by the EpiQuik^™^ m6A RNA Methylation Quantification Kit (Colorimetric) (P-9005, EpiGentek, USA), according to the manufacture’s instruction.

### Small interfering RNAs (siRNAs) and Plasmids

pCMV3-Flag-FTO, pCMV3-empty vector, pCMV-Myc-Mettl3 plasmids were obtained from SinoBiological (Beijing, China). siRNA against FTO (si-FTO) or Fam-labelled si-FTO (sense:5’- GCACCUACAAGUACUUGAAdTdT-3’, and anti-sense 5’- UUCAAGUACUUGUAGGUGCdTdT-3’), and siRNA control (sense:5’- UUCUCCGAACGUGUCACGUdTdT-3’, and anti-sense 5’- ACGUGACACGUUCGGAGAAdTdT-3’) were synthetized by Guanghzhou Igebio Co., Ltd. siRNAs or plasmids were transfected into MN9D dopaminergic neurons using Exfect Transfection Reagent (Vazyme Biotech, China), as our described previously [23].

### Quantitative Real-Time PCR (qRT-PCR)

Total RNA was extracted from the substantia nigra in PD mice or MN9D cells, and inversely transcribed into cDNA. Then, RT-PCR was performed, as described previously [23]. Primer sequences were shown in the Additional file 1: Table S1.

### Western blot analysis

Western Blot was performed as our previously described [20]. The primary antibodies include Mettl14 (220030, Abcam, USA), FTO (27226-1-AP, Proteintech, China), α-syn (10842-1-AP, Proteintech, China), TH antibody (25859-1-AP, Proteintech, China), ATM antibody (27156-1-AP, Proteintech, China), YTHDF3 antibody (25537-1-AP, Proteintech, China), CD81 antibody (27855-1-AP, Proteintech, China), Syntenin (ab19903, Abcam, USA), HSP90B1 antibody (14700-1-AP, Proteintech, China), Calnexin antibody (10427-2-AP, Proteintech, China), TSG101 antibody (28283-1-AP, Proteintech, China), and GAPDH antibody (60004-1-AP, Proteintech, China).

### Bioinformatics analysis

The potential m6A modification site on ATM mRNA were analyzed by m6AVar (http://m6avar.renlab.org/). Data was downloaded from the Gene Expression Omnibus (GEO) database (http://www.ncbi.nlm.nih.gov/geo). GSE54282 database included the gene expression profiles of striatum regions in 6 healthy persons and 6 PD patients. The differentially expressed genes from GSE54282 were analyzed using the limma package [24]. |LogFC|> 1.5 and P value < 0.05 were used as threshold for nominally significant differential expression. ggplot2 and pheatmap packages were used to draw the volcanic maps and heat maps, respectively. Pathway and process enrichment analysis were performed with Metascape (http://metascape.org/gp).

### DiD labeling and cellular tracking of exosomes

Isolated exosomes were labelled with the lipid membrane dye DiD (Sigma, USA). Briefly, exosomes (50 µg) were first stained with 12 µM DiD for 30 min in the dark. Then, DiD-labeled exosomes were incubated with MN9D cells for 24 h. Subsequently, MN9D cells were stained with FITC-Phalloidin (40735ES75, YEASEN, China), and then imaged by confocal microscopy (LSM800, Zeiss, Germany).

### Loading si-FTO or Fam-labelled si-FTO into exosomes

si-FTO or Fam-labelled si-FTO were loaded into exosomes through sonication, as our previously described [25]. Briefly, 300 nM si-FTO or Fam-labelled si-FTO were mixed with 100 ng of exosomes, and then sonicated by a Model 505 Sonic Dismembrator: 20% amplitude, 6 cycles of 30 s on/off with a 30 s cooling period between each cycle. Later, the mixture was incubated at 37 °C for 60 min to restore the integrity of the membrane of exosomes. Excess free siRNAs were removed by ultracentrifugation at 120,000 g for 2 h.

### Detection of the distribution of exosomes loaded with siRNAs

PD mice were randomly assigned to three groups with 6 mice per group (PD group, PD + exosomes-si-Control group, PD + exosomes-si-FTO group). Control mice (n = 6) were injected with equal volume of 0.9% saline. 24 h after the last injection of MPTP, PD mice were intravenously injected with DiD-loaded exosomes containing Fam-labelled siRNAs (100 ul/day) for 3 consecutive days. Then, the mice were sacrificed and the brain tissues were collected and frozen in cryosection tissue-Tek-OCT. Frozen tissues were then sectioned with a thickness of 10 μm and fixed with acetone at −20 °C. After washing with PBS, sections were stained with DAPI (Solarbio) and visualized by confocal laser scanning microscopy (LSM800, Zeiss, Germany).

### Statistical analysis

Data were analyzed using the GraphPad Prism 5.0 software (GraphPad Software Inc.) Differences between groups were analyzed by the Student’s *t*-test or one-way analysis of variance (ANOVA)/Bonferroni multiple-comparison post hoc test. *P*-values (represented by asterisks), where *p < 0.05; **p < 0.01.

## Results

### The m6A level of dopaminergic neurons significantly reduced in PD models in vitro and in vivo

**Fig. 1:**
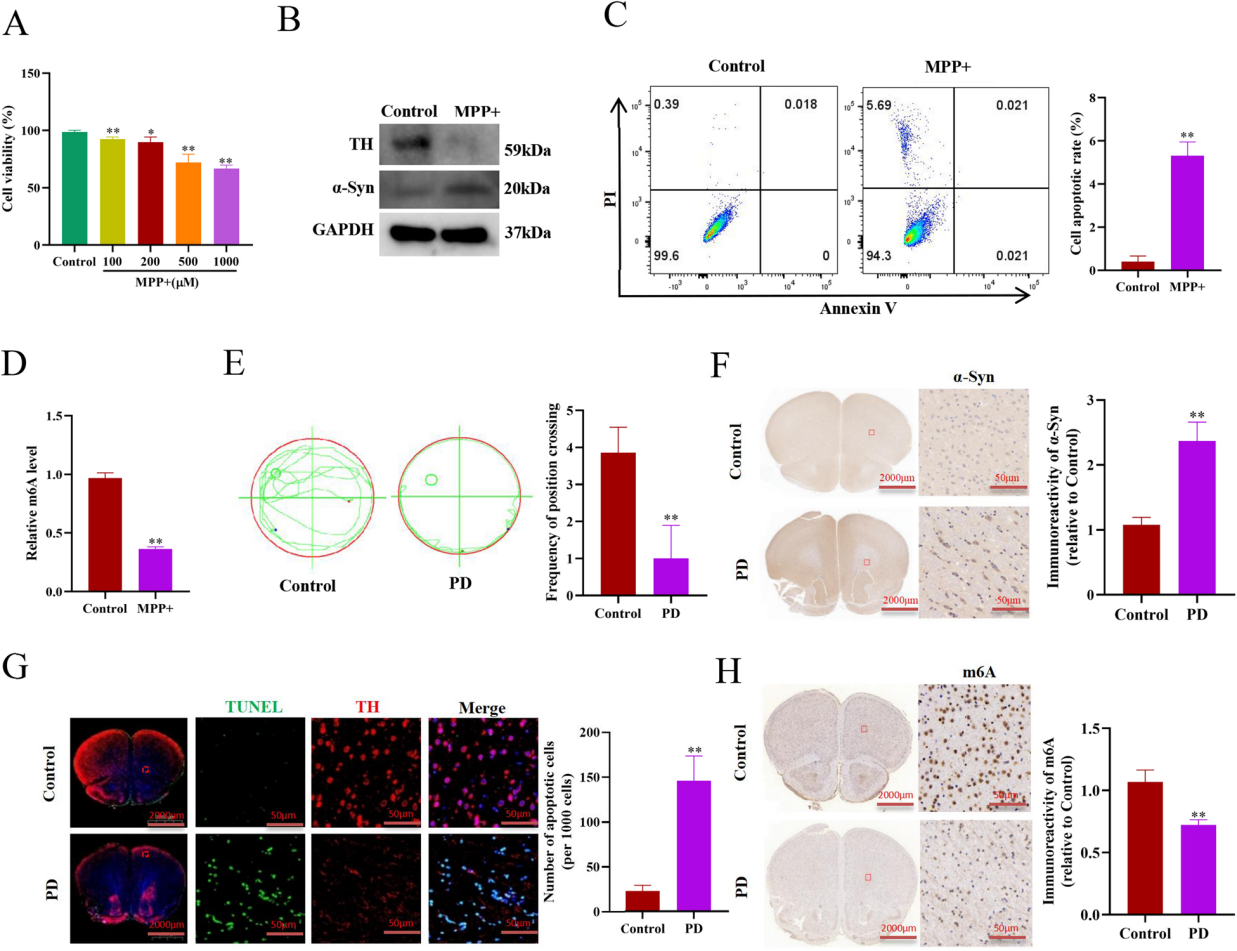
The m6A level of dopaminergic neurons significantly reduced in PD models in vitro and in vivo. **A** Cell viability of mouse MN9D dopaminergic neurons treated with different concentrations of MPP + for 24 h (n = 3). **B** Western blotting analysis of TH and α-Syn protein expressions in the MN9D dopaminergic neurons treated with 500 μM MPP + for 24 h. **C** Flow cytometry analysis of apoptosis of MN9D dopaminergic neurons treated with 500 μM MPP + or not for 24 h (n = 3). **D** Detection of the total RNA m6A methylation levels in the MN9D dopaminergic neurons treated with 500 μM MPP + for 24 h (n = 3). **E** Morris water maze assay analysis of the Control and PD mice (n = 6). **F** IHC analysis of α-Syn in the striatum of mice PD model (n = 3). **G** Immunofluorescence staining analysis of dopaminergic neuronal apoptosis in the striatum of mice PD model (n = 3). **H** IHC analysis of m6A in the striatum of mice PD model (n = 3). Data are represented as mean ± SD, *represents P < 0.05, **represents P < 0.01

To explore the changes of m6A modification in PD, MPP + -stimulated mouse dopaminergic neuronal MN9D cells were used to establish an in vitro PD model. As shown in Fig. 1A, MPP + treatment gradually reduced the cellular viability in a dose-dependent manner, and 500 μM MPP + was used in the following in vitro studies. As expected, α-Syn protein expression was significantly increased (Fig. 1B), tyrosine hydroxylase expression, a specific maker of dopaminergic neurons, was evidently decreased (Fig. 1B), and dopaminergic neuronal death was notably increased in the cellular PD model (Fig. 1C), compared to control. Furthermore, we found the total m6A level was evidently decreased in the cellular PD model (Fig. 1D).

Subsequently, a mice PD model was established by intraperitoneal injection of MPTP to C57BL/6 mice. As shown in Fig. 1E, the spatial learning and memory capacity of PD mice significantly declined, detected by the morris water maze test. Furthermore, IHC staining showed that α-Syn expression was significantly increased in the striatum of PD group (Fig. 1F), and immunofluorescent staining showed dopaminergic neurons died in the striatum of PD group (Fig. 1G). In addition, IHC staining showed that the total m6A level was significantly downregulated in the striatum of PD mice, compared to control (Fig. 1H). Taken together, these data demonstrate the m6A level of dopaminergic neurons significantly reduced in PD models in vitro and in vivo.

### m6A demethylase FTO significantly increased in PD models in vitro and in vivo

**Fig. 2:**
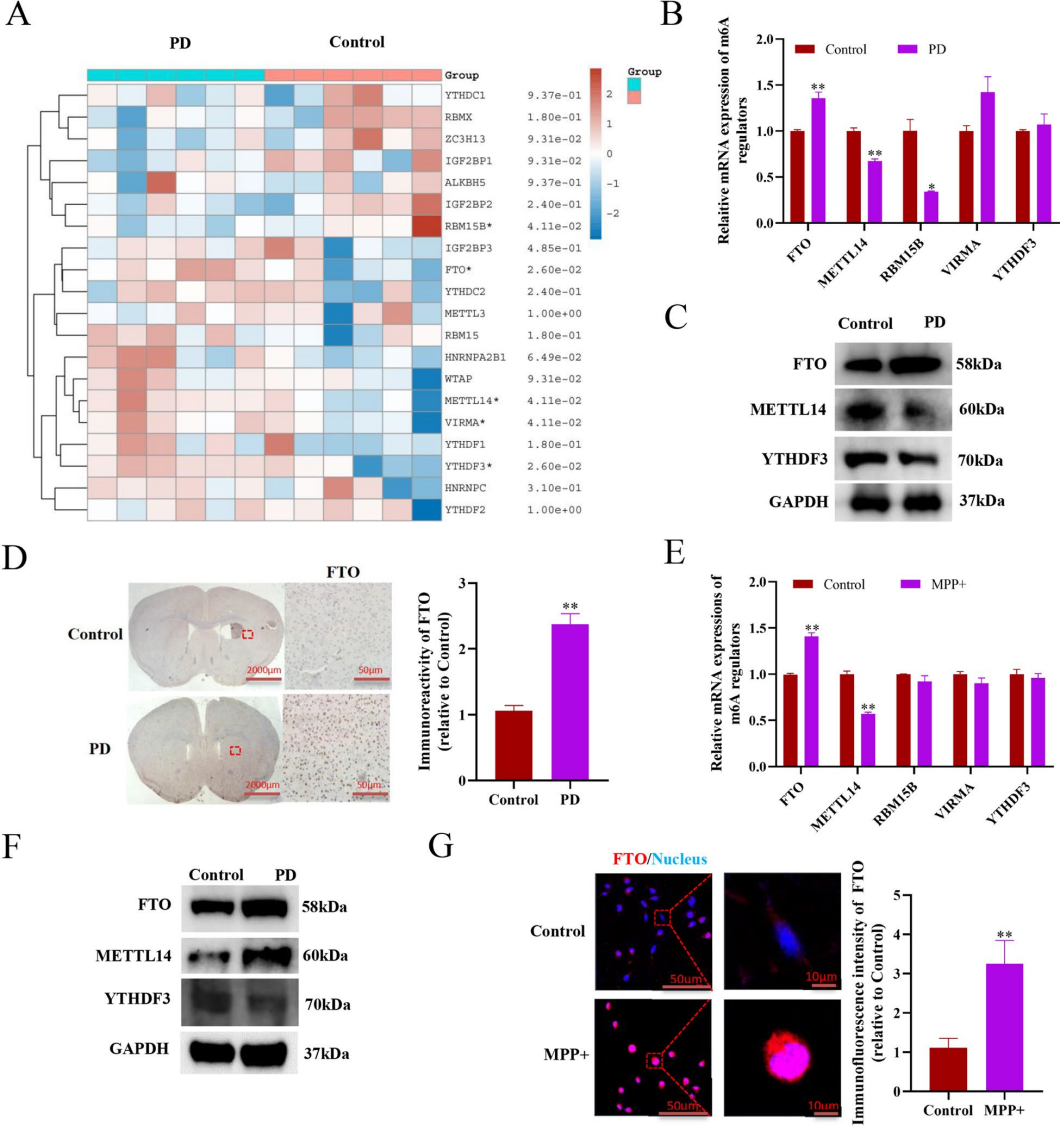
m6A demethylase FTO significantly increased in PD models in vitro and in vivo. **A** Heatmap of the m6A-related genes in the striatum of 6 cases of PD patients and 6 cases of healthy persons from GSE54282 datasets. **B** qRT-PCR analysis of m6A-related genes in the striatum of mice PD model (n = 3). **C** Western blotting analysis of the indicated protein expressions in the striatum of mice PD model. **D** IHC analysis of FTO in the striatum of mice PD model (n = 3). **E** qRT-PCR analysis of m6A-related genes in the cellular PD model (n = 3). **F** Western blotting analysis of the indicated protein expressions in the cellular PD model. **G** Immunofluorescence staining analysis of FTO expressions in the cellular PD model (n = 3). Data are represented as mean ± SD, *represents P < 0.05, **represents P < 0.01

Next, we analyzed the differential expression of those m6A modification-related genes in the striatum tissues from 6 PD patients and 6 healthy individuals from the GSE54282 datasets [26], and found the m6A demethylase fat mass and obesity-related protein (FTO) and the m6A reader YTH domain family protein 3 (YTHDF3) expressions were significantly increased, but the methylase RNA binding motif protein 15B (RBM15B), the methyltransferase-like 14 (Mett14), and the vir-like m6A methyltransferase associated (VIRMA) were significantly decreased in the PD patients, compared to healthy individuals (Fig. 2A). Furthermore, we detected the mRNA expressions of the above five genes in PD models in vivo and in vitro, and found the significant upregulation of FTO and downregulation of Mett14 in PD models (Fig. 2B, E). Moreover, Western blotting analysis showed FTO protein expression indeed significantly increased in PD models in vivo and in vitro (Fig. 2C, F). In addition, IHC and immunofluorescent staining analysis also confirmed FTO was aberrantly upregulated in PD models in vivo and in vitro (Fig. 2D, G)*.*

### Knockdown of FTO inhibits the cellular death-related factor ataxia telangiectasia mutated (ATM) expression and alleviates dopaminergic neuronal death in PD models in vitro

**Fig. 3:**
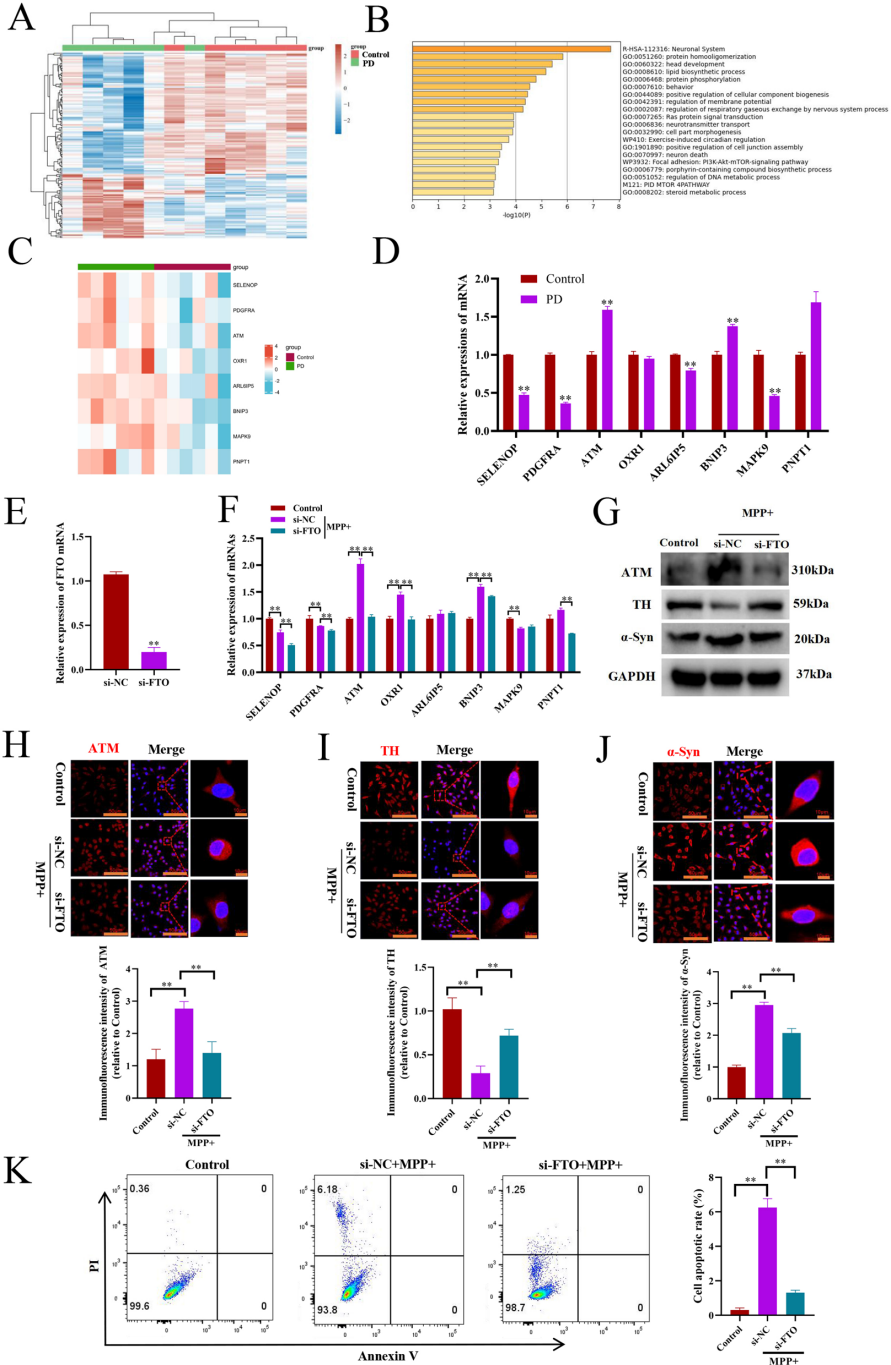
Knockdown of FTO inhibits ATM expression and alleviates dopaminergic neuronal death in PD models in vitro. **A** Heatmap of the differentially expressed genes in the striatum of 6 cases of PD patients and 6 cases of healthy persons from GSE54282 datasets. **B** The pathway and process enrichment analysis of the significantly upregulated genes through Metascape. **C** Heatmap of the significantly upregulated genes which are involved in neuronal death. **D** qRT-PCR analysis of the indicated genes in the mice PD model (n = 3). **E** qRT-PCR analysis of FTO mRNA expressions in MN9D dopaminergic neurons transfected with si-NC or si-FTO (n = 3). **F** qRT-PCR analysis of the indicated genes in MN9D dopaminergic neurons transfected with si-NC or si-FTO and treated with MPP + (n = 3). **G** Western blotting analysis of the indicated protein expressions in MN9D dopaminergic neurons transfected with si-NC or si-FTO and treated with MPP + . **H**, **I** and **J** Immunofluorescence staining analysis of ATM, TH, and α-Syn expressions in MN9D dopaminergic neurons transfected with si-NC or si-FTO and treated with MPP + (n = 3). **K** Flow cytometry analysis of apoptosis of MN9D dopaminergic neurons transfected with si-NC or si-FTO and treated with MPP + for 24 h (n = 3). Data are represented as mean ± SD, *represents P < 0.05, **represents P < 0.01

To further investigate the effect of FTO on dopaminergic neuronal death in PD, we firstly analyzed the differentially expressed genes in the striatum between PD patients and healthy individuals from GSE54282 datasets, and found 176 genes upregulated and 53 genes downregulated (Fig. 3A). We then performed the pathway and process enrichment analysis on the 176 upregulated genes through Metascape, and found these genes were mainly enriched in protein homooligomerization, lipid biosynthetic process, neuron death, and so on (Fig. 3B). Furthermore, 8 genes, including selenoprotein P (SELENOP), platelet-derived growth factor receptor alpha (PDGFRA), ataxia telangiectasia mutated (ATM) kinase, oxidation resistance 1 (OXR1), ADP ribosylation factor-like GTPase 6 interacting protein 5 (ARL6IP5), BCL2 Interacting Protein 3 (BNIP3), mitogen-activated protein kinase 9 (MAPK9), and Polyribonucleotide Nucleotidyltransferase PNPase 1 (PNPT1) were identified to be involved in neuron death (Fig. 3C).

Subsequently, we detected the mRNA expression of the above 8 genes in PD models in vitro and in vivo, and found that SELENOP, PDGFRA and MAPK9 mRNA expressions were significantly decreased in both cellular and animal PD models, whilst both ATM and BNIP3 were significantly increased (Fig. 3D, F). Interestingly, knockdown of FTO significantly suppressed ATM expression in MPP + stimulated MN9D cells. (Fig. 3E, F).

Considering that the activation of ATM, an important regulator of the DNA damage response, induces cell apoptosis [27, 28], and inhibition of ATM reduces the severity of Huntington’s disease in both cellular and animal models [29], we focused on ATM in the following studies. Moreover, knockdown of FTO significantly inhibited the protein expressions of ATM and α-Syn, but increased TH protein in MPP + -treated MN9D cells (Fig. 3G–J). In addition, as expected, knockdown of FTO evidently alleviated dopaminergic neuronal death in PD in vitro (Fig. 3K)*.*

### FTO promotes ATM expression through m6A-dependent stabilizing of ATM mRNA in dopaminergic neurons

**Fig. 4:**
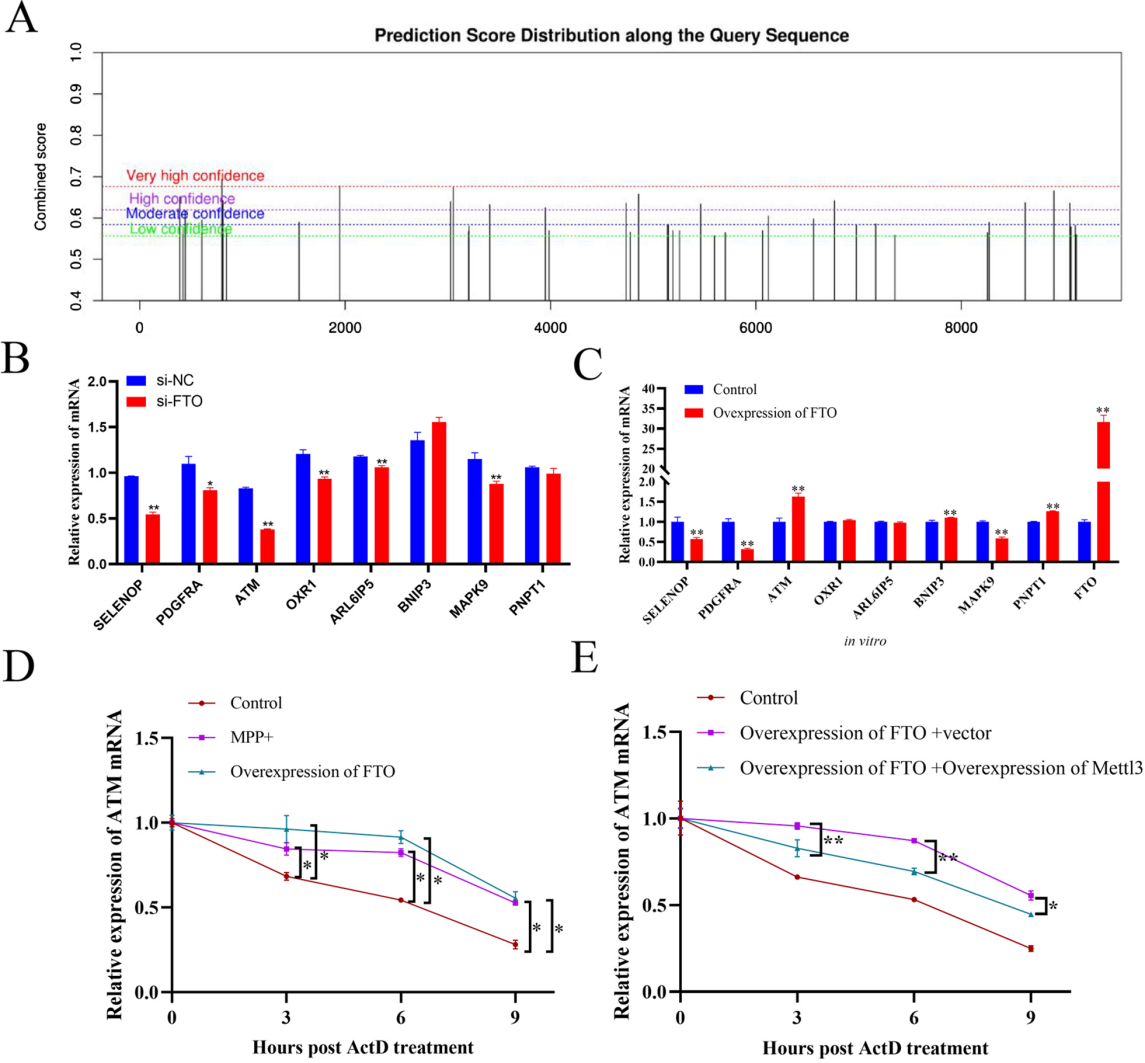
FTO promotes ATM expression through m6A-dependent stabilizing of ATM mRNA in dopaminergic neurons. **A** The potential m6A sites on ATM mRNA were analyzed by m6Avar. **B** qRT-PCR analysis of the indicated genes in MN9D dopaminergic neurons transfected with si-NC or si-FTO (n = 3). **C** qRT-PCR analysis of the indicated genes in MN9D dopaminergic neurons transfected with the empty control plasmid or pCMV-Flag-FTO plasmid (n = 3). **D** qRT-PCR analysis of ATM mRNA in MN9D dopaminergic neurons transfected with pCMV-Flag-FTO plasmid or treated with MPP + (n = 3). **E** qRT-PCR analysis of ATM mRNA in MN9D dopaminergic neurons co-transfected with pCMV-Flag-FTO plasmid + pCMV-Myc vector or pCMV-Flag-FTO plasmid + pCMV-Myc-Mettl3 (n = 3). Data are represented as mean ± SD, *represents P < 0.05, **represents P < 0.01

Subsequently, we investigated the underlying mechanism by which FTO regulates ATM expression. Through SRAMP analysis, two potential m6A modification sites were identified at 799 and 1749 positions of ATM mRNA with very high confidence (Fig. 4A). We then examined the effects of FTO on ATM mRNA expression, since m6A modification regulates mRNA splicing, stability, and translation [6]. As shown in Fig. 4B, C, overexpression of FTO significantly promoted ATM mRNA expression, whereas knockdown of FTO significantly reduced ATM expression in dopaminergic neurons. Furthermore, either MPP + treatment or overexpression of FTO in MN9D cells significantly enhanced ATM mRNA stability (Fig. 4D). Interestingly, co-overexpression of the m6A methylase Mettl3 suppressed the effect of FTO overexpression on ATM mRNA stability in dopaminergic neurons. (Fig. 4E). Collectively, these results suggest that FTO upregulates ATM expression through increasing its mRNA stability in dopaminergic neurons.

### Exosomal delivery of si-FTO (Exo-si-FTO) efficiently suppressed FTO expression and dopaminergic neuronal death in PD model in vitro

**Fig. 5:**
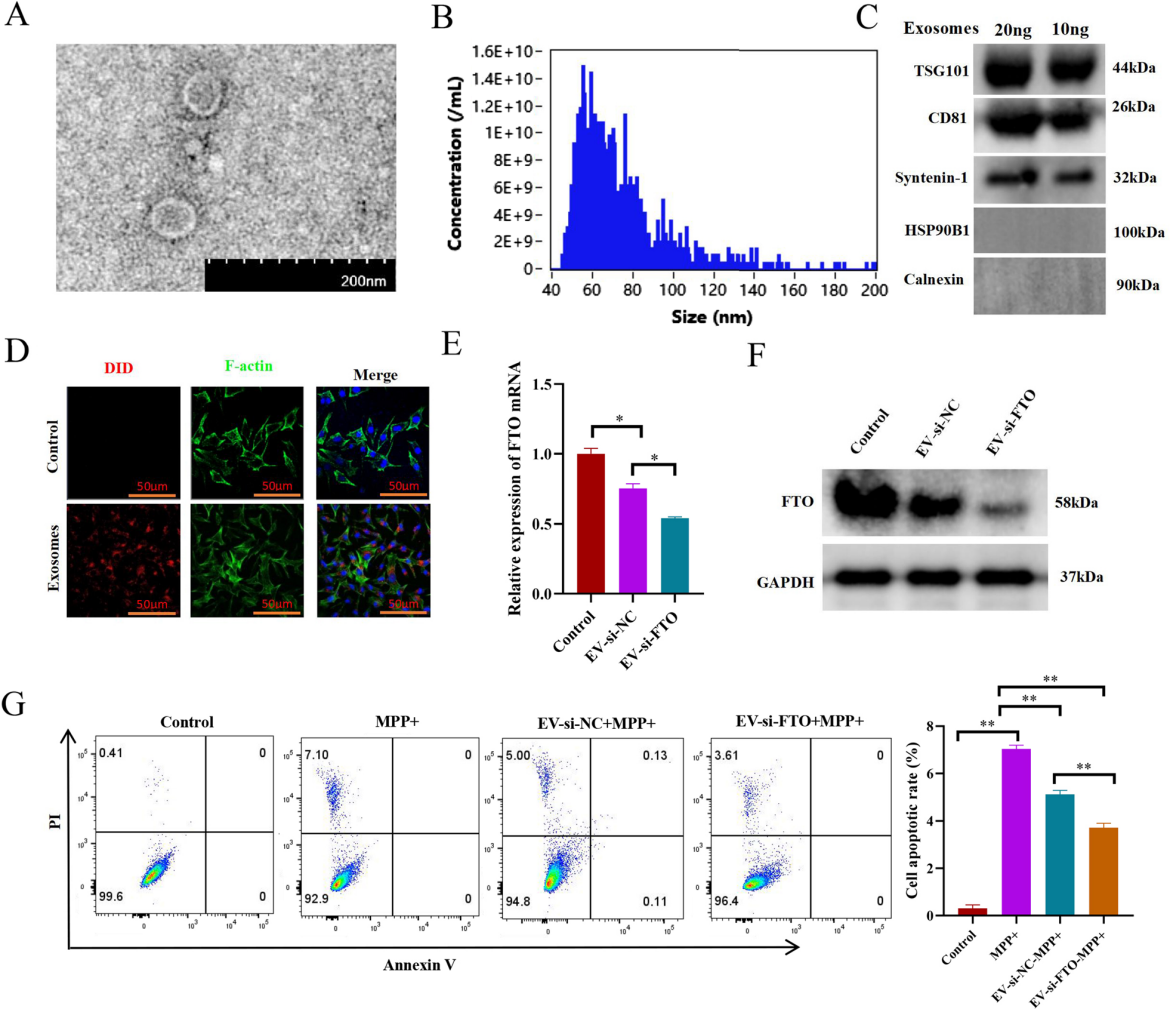
Exo-si-FTO efficiently suppressed FTO expression and dopaminergic neuronal death in PD model in vitro. **A** Electron microscope scanning of exosomes from MSCs. **B** Nanoparticle tracking analysis of exosomes from MSCs. **C** Western blotting analysis of TSG101,CD81, Synthenin-1, HSP90B1 and Calnexin protein expressions in exosomes from MSCs. **D** Confocal microscopy analysis of the cellular distributions of exosomes which was stain with DID (red) in MN9D dopaminergic neurons. The cellular morphology was stained with FITC-Phalloidin (Green). **E** qRT-PCR analysis of FTO mRNA expression in MN9D dopaminergic neurons incubated with MSC-exo-si-NC or MSC-exo-si-FTO (n = 3). **F** Western blotting analysis of FTO expression in MN9D dopaminergic neurons incubated with MSC-exo-si-NC or MSC-exo-si-FTO. **G** Flow cytometry analysis of apoptosis of MN9D dopaminergic neurons incubated with MSC-exo-si-NC or MSC-exo-si-FTO, in the presence of MPP + (n = 3). Data are represented as mean ± SD, *represents P < 0.05, **represents P < 0.01

Generally, the therapeutic effect of neural medicine is greatly hampered by the blood–brain barrier (BBB) [30], and MSC-exo could permeate the BBB and be potentially utilized as an efficient and safe drug delivery system for neural disease therapies [31]. Therefore, we next explored to whether MSC-exo could be harnessed to deliver si-FTO for PD treatment. Firstly, exosomes were isolated and purified from serum-free medium conditioned by MSCs. As shown in Fig. 5A, the isolated exosomes were membrane-enclosed vesicles, detected by transmission electron microscopy (TEM). Furthermore, the diameter of isolated exosomes was about 40–150 nm by the nanoparticle flow cytometry analysis (Fig. 5B). Moreover, Western blotting analysis showed the expressions of exosomal biomarkers TSG101 CD81 and Synteinin-1, but not the endoplasmic reticulum biomarkers HSP90B1 and Calnexin, in isolated products (Fig. 5C), confirming the successful preparation of MSC-exo.

Then, the isolated exosomes were labelled with DiD dye, and incubated with dopaminergic MN9D cells. As shown in Fig. 5D, DiD-labelled exosomes (Exo-DiD, red) were readily endocytosed by MN9D cells and distributed around nuclei, indicating their suitability for developing neural disease therapies. Next, the si-FTO and si-NC were encapsulated into exosomes by sonication, and designated as Exo-si-FTO and Exo-si-NC, respectively. Subsequently, Exo-si-FTO and Exo-si-NC were used to treat MN9D cells to examine the inhibitory effects on FTO expression. As shown in Fig. 5E, F, surprisingly, both Exo-si-NC and Exo-si-FTO showed significant inhibitory effects on FTO mRNA and protein expression, although the latter worked better. Moreover, both Exo-si-NC and Exo-si-FTO significantly alleviated dopaminergic neuronal death in PD in vitro, and the latter performed better protectively effects (Fig. 5G).

### Synergistic si-FTO loading of exosomes effectively protect dopaminergic neuronal death in PD model in vivo

**Fig. 6:**
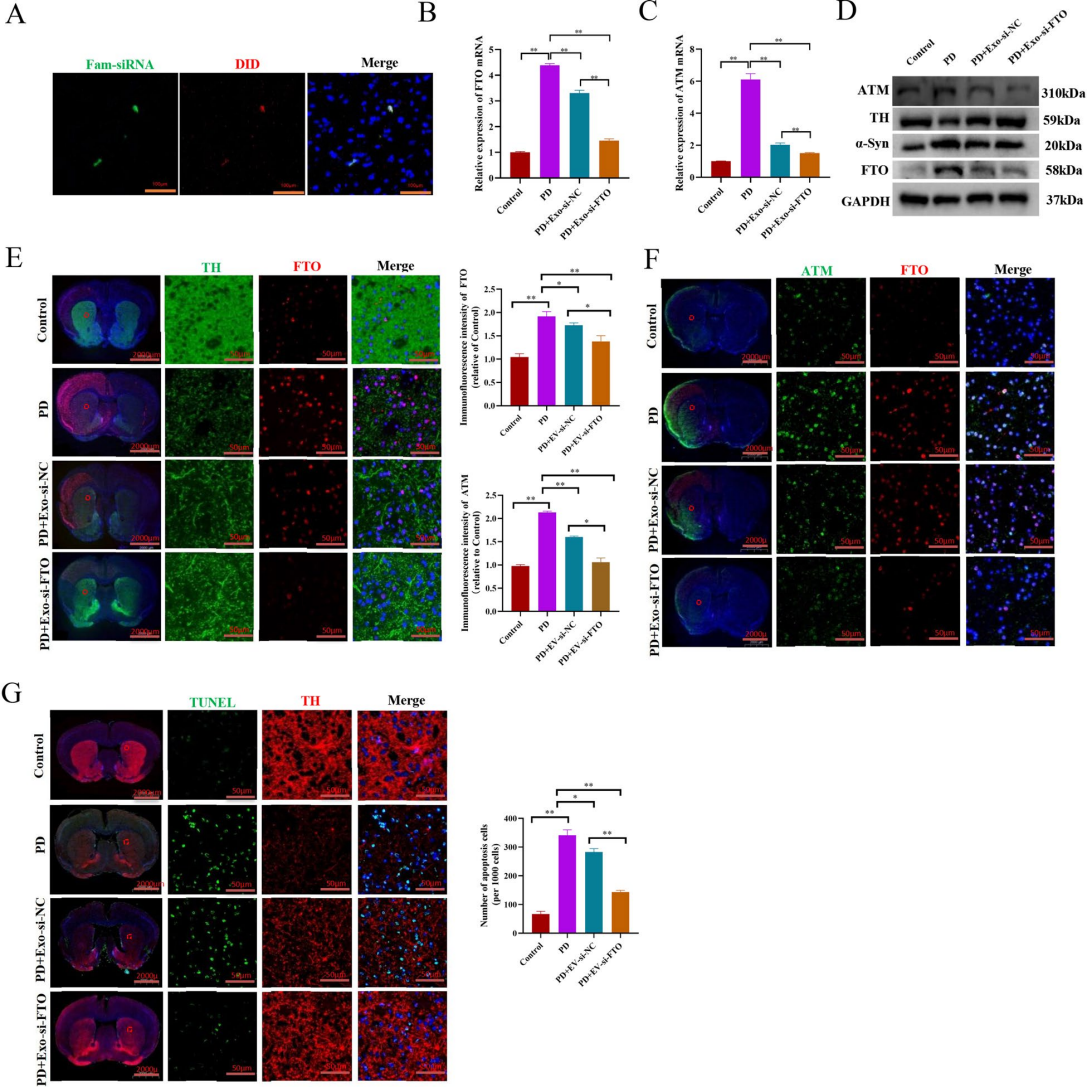
Synergistic si-FTO loading of exosomes effectively protect dopaminergic neuronal death in PD model in vivo. **A** Confocal microscopy analysis of the distributions of MSC-exo (red)-si-FTO (green) in the striatum of mice PD model. **B** and **C** qRT-PCR analysis of FTO and ATM mRNA expressions in the striatum of mice PD model injected with MSC-exo-si-NC or MSC-exo-si-FTO (n = 3). **D** Western blotting analysis of TH, α-Syn, ATM and FTO protein expressions in the striatum of mice PD model injected with MSC-exo-si-NC or MSC-exo-si-FTO. **E** Immunofluorescence staining analysis of FTO and TH expressions in the striatum of mice PD model injected with MSC-exo-si-NC or MSC-exo-si-FTO (n = 3). **F** Immunofluorescence staining analysis of FTO and ATM expressions in the striatum of mice PD model injected with MSC-exo-si-NC or MSC-exo-si-FTO (n = 3). **G** Immunofluorescence staining analysis of the apoptosis of dopaminergic neurons in the striatum of mice PD model injected with MSC-exo-si-NC or MSC-exo-si-FTO (n = 3).Data are represented as mean ± SD, *represents P < 0.05, **represents P < 0.01

Subsequently, Fam-labelled si-FTO or si-NC was loaded into DiD-stained exosomes to prepare double-labelled exosomes, and the obtained product was designated as Fam-DiD-Exo, which was utilized to evaluate the potential of exosomal delivery of siRNA for PD treatment in the murine model. Then, PD mice were intravenously injected with Fam-DiD-Exo (100 µL/mouse/daily, 1200 nmol/kg, daily) for 3 consecutive days. After 24 h, the mice brain tissues were collected and examined for the bio-distribution of double-labelled exosomes by laser scanning confocal microscopy. As shown in Fig. 6A, DiD and Fam fluorescent signals were co-localized in the striatum site within brain, indicating that the administered exosomes have permeated the BBB and successfully delivered si-FTO into the neural damaged site.

Importantly, the Exo-si-FTO therapy successfully reduced FTO expression, resulting in significantly reduced expression of both ATM and α-Syn but increased expression of TH in the brain of PD mice (Fig. 6B–D), indicating that exosomal delivery of si-FTO is a highly effective strategy for PD treatment. Interestingly, the Exo-si-NC administration also significantly decreased FTO, ATM and α-Syn expression levels, but increased TH expression (Fig. 6B–D), showing protective effect on PD as well, despite with relatively lower effectiveness than Exo-si-FTO, indicating that MSC-exo alone may also modulate the m6A modification level in vivo. Moreover, immunofluorescence staining of animal brain sections further confirmed the results of Fig. 6D (Fig. 6E, F). Finally, immunofluorescent staining showed Exo-si-FTO evidently inhibited dopaminergic neuronal death in PD model in vivo (Fig. 6G).

Collectively, these data show that both MSC-exosomes and Exo-si-FTO treatments presented therapeutic benefits on PD in vivo and in vitro, however, the latter was relatively much more effective.

## Discussion

This study for the first time reveals the significant role of the m6A demethylase FTO in regulating dopaminergic neuronal death in PD. FTO was evidently increased in dopaminergic neurons in PD models in vitro and in vivo. Importantly, knockdown of FTO significantly inhibited α-Syn expression and dopaminergic neuron apoptosis, and increased TH protein through promoting pro-apoptotic protein ATM expression via m6A-dependent stabilization of its mRNA. Besides, just as important, our study innovatively employed MSC-derived exosomes as drug delivery vehicles to load si-FTO for PD therapy. It is inspiring that Exo-si-FTO could sufficiently cross the BBB, and inhibit dopaminergic neuron apoptosis. Therefore, FTO may serve as a potential therapeutic target for PD, and MSC-derived exosomes loaded with si-FTO may be as an effective therapeutic agent for PD treatment.

M6A is highly abundant in the brain and plays important roles in neurological development and its dysregulation is involved in several neurodegenerative diseases [8, 9]. For example, aberrant level of m6A modification leads to hippocampal memory defects in a murine model of Huntington’s disease [32]. Global m6A modification level was significantly downregulated in both 6-OHDA-exposed PC12 cellular model and the striatum of PD rats [33]. Consistent with previous study [33], our study found that the total m6A level significantly decreased and the m6A demethylase FTO protein expression evidently increased in PD models using a MPTP-treated mice model and a MPP + -induced MN9D cellulal model, respectively, suggesting the dysregulation of m6A modification may be crucially involved in the pathogenesis of PD. Furthermore, our study identifies FTO promotes dopaminergic neuronal death in PD through regulating ATM mRNA stability in an m6A-dependent manner, which deepens our understanding on the regulatory role of FTO in PD and supplies a potential target for the development of therapeutic drug.

The protein kinase ATM, known as a DNA repair protein, acts important roles in maintaining genome integrity [27]. Following damage-sensing Mre11, Rad50, and Nbs1 (MRN) complex in conjunction with DNA double-strand breaks, ATM is activated to phosphorylate numerous of substrates such as KAP1, Akt, and p53 to regulate diverse cellular processes, including DNA repair, cell apoptosis, and so on [34–36].In Huntington’s disease, ATM is found to trigger mitochondrial-mediated apoptosis through activation of AMP-activated protein kinase (AMPK) [37]. Similarly, ATM is found to be specifically activated in the dopaminergic neurons to induce apoptosis in synucleinopathy models of PD [38, 39]. Our study for the first time reports FTO positively regulates ATM expression through m6A-mediated mRNA stability, and knockdown of FTO evidently suppresses ATM expression and improves dopaminergic neuronal death in PD model in vivo and in vitro*.* Our study indicates a novel regulation axis of FTO-m6a-ATM-neuronal death in PD. However, the detailed regulatory mechanism underlying FTO regulating ATM expression still needs further investigation in the future. In addition, we also found that BNIP3 mRNA was significantly increased in PD models in vitro and in vivo, and knockdown of FTO significantly suppressed BNIP3 expression in vitro*.*BNIP3 is a pro-apoptotic protein, which disrupts mitochondrial membrane potential and induces mitochondrial apoptosis [40]. BNIP3 has been found to be involved in cardiomyocyte apoptosis during heart failure and in doxorubicin-induced cardiomyocyte pyroptosis [41, 42]. We also identified the potential m6A modification sites with high confidence in BNIP3 mRNA (data not shown). Whether BNIP3 is also involved in m6A-mediated dopaminergic neuronal death in PD needs to be further investigated in the following studies.

Exosomes, which contains an abundance of proteins, miRNA, and so on, act important roles for cell–cell communication [43, 44]. For example, exosome-mediated miR-155 transfer from smooth muscle cells to endothelial cells induces endothelial injury and promotes atherosclerosis [45]. Cardiomyocyte-derived exosomal miR-92a promotes myofibroblast activation after myocardial infarction [46]. Glioblastoma multiforme-associated macrophages (GAMs)-derived exosomes promote glioblastoma multiforme cells’ resistance against temozolomide via secreting oncomiR-21 [47]. T cells-derived exosomal miR-142-3p augments vascular permeability through down-regulation of endothelial RAB11FIP2 expression during cardiac allograft rejection [48]. Chemoresistant breast cancer cells promote the phenotype of sensitive cells to chemoresistance via exosome-mediated miR-155 transfer [49]. Exosomes derived from cardiomyocytes promote myofibroblast phenoconversion after myocardial infarction via miR-195 transfer [50]. miR-155-5p in serum derived-exosomes promotes ischaemia–reperfusion injury by reducing CypD ubiquitination by NEDD4 [51]. Besides miRNAs, exosomes also induce N-glycan remodeling in recipient cells via N-acetylglucosaminyltransferase-V transfer [44]. Nowadays, therapeutic miRNA delivery via exosomes has attracted more and more attention [52]. Human adipose tissue-derived mesenchymal stromal/medicinal signaling cells-derived exosomes-encapsulated miR-125b inhibits hepatocellular carcinoma cell proliferation [53]. Localized injection of miR-21-enriched extracellular vesicles effectively restores cardiac function after myocardial infarction [54]. In this study, intravenous injectionof MSC-derived exosomes-encapsulated si-FTO sufficiently crossed the BBB, and inhibited dopaminergic neuron apoptosis in an in vivo PD model. Interestingly, our study found that alone MSC-derived exosome treatment could significantly suppress α-Syn expression, and increase TH expression in both cellular and animal PD models, confirming the great potential of MSC-exo for PD therapies, which is consistent with previous studies [55, 56]. Unexpected, we found MSC-exo treatment alone also evidently suppressed FTO expression in PD models in vivo and in vitro, suggesting the benefits of exosome therapy may be partially mediated via m6A modification. Interestingly, human MSC-exosomal miR-627-5p has been demonstrated to ameliorate non-alcoholic fatty liver disease via downregulating FTO expression [57], which indicates the inhibitory effect of FTO expression by MSC-exosomes may be mediated via exosomal miR-627-5p as well in this study. However, this needs further investigation in the following studies.

Although our study innovatively employed MSC-derived exosomes as drug delivery vehicles to load si-FTO for PD therapy, there are still some limitations for this study. First, si-RNAs were loaded to exosomes via sonication in this study, and the si-RNA loading efficiency is low. We would try to improve the loading efficiency of exomoses with thin-film hydration developed by Sruti Bheri et al. [58]. Second, we only detected the distribution of exosomes in the brain tissue. How does the distribution of exosomes in other organs needs to be deeply investigated in the following studies. The third, despite that exosomes could cross the BBB into brian in the study, the exosomes crossing efficiency still needs to be improved in the following studies.

In conclusion, our study reveals that the m6A demethylase FTO promotes dopaminergic neuronal death in PD via m6A-dependent regulation of ATM mRNA. Besides, MSC-exo could be employed to deliver si-FTO into the striatum of PD mice, which further enhances the therapeutic efficacy of exosomes for PD treatment. Therefore, MSC-exosomal delivery of si-FTO potentially constitutes a highly effective novel therapy for PD.

### Supplementary Information

**Additional file 1:** **Table S1.** The primers sequences of qRT-PCR.

## Data Availability

The datasets used or analysed during the current study are available from the corresponding author on reasonable request.

## Abbreviations

PD: Parkinson's disease
m6A: N6-methyladenosine
MSC: Mesenchymal stem cells
α-Syn: Synuclein
ATM: Ataxia telangiectasia mutated
Mettl3: Methyltransferase-like 3
TH: Tyrosine hydroxylase
MPTP: 1-Methyl-4-phenyl-1,2,3,6-tetrahydropyridine
TEM: Transmission electron microscop
MPP +: 1-Methyl-4-phenylpyridinium
IHC: Immuno-Histochemistry
qRT-PCR: Quantitative Real-Time PCR
SELENOP: Selenoprotein P
PDGFRA: Platelet-derived growth factor receptor alpha
OXR1: Oxidation resistance 1
ARL6IP5: ADP ribosylation factor-like GTPase 6 interacting protein 5
BNIP3: BCL2 Interacting Protein 3
MAPK9: Mitogen-activated protein kinase 9
PNPT1: Polyribonucleotide Nucleotidyltransferase PNPase 1
