# Supplementary material for: FTO-targeted siRNA delivery by MSC-derived exosomes synergistically alleviates dopaminergic neuronal death in Parkinson's disease via m6A-dependent regulation of ATM mRNA
Source: J Transl Med. 2023 Sep 22;21:652. doi: 10.1186/s12967-023-04461-4 (PMC10515429; doi:10.1186/s12967-023-04461-4)
Supplement: Supplementary file 1 — Additional file 1: Table S1. The primers sequences of qRT-PCR. [file 12967_2023_4461_MOESM1_ESM.docx]

Additional Table 1. The primers sequences of qRT-PCR.

| Gene | Sense | Squence (5’-3’) |
| --- | --- | --- |
| α-syn | F | CCGCACATTTGCCCAGTTC |
|  | R | TGCACCGTAAGCCTTCAGCTC |
| TH | F | AAGACTATGAGCCTGAAGCCTAAG |
|  | R | AGTGTGAAGCCACAACAATATCC |
| FTO | F | TTCATGCTGGATGACCTCAATG |
|  | R | GCCAACTGACAGCGTTCTAAG |
| METTL14 | F | CTGAGAGTGCGGATAGCATTG |
|  | R | GAGCAGATGTATCATAGGAAGCC |
| RBM15B | F | AGGGCGAAGGTGGCTATGT |
|  | R | GCGAGGTGTTAGGTCCGAG |
| VIRMA | F | ATGTCATGGAAACTGCACCTC |
|  | R | GAGTGCTGAAAACCAAACCCA |
| YTHDF3 | F | CATAGGGCAACAGAGGAAACAG |
|  | R | ATCTCCAGCCGTGGACCAT |
| ATM | F | GATCTGCTCATTTGCTGCCG |
|  | R | GTGTGGTGGCTGATACATTTGAT |
| SELENOP | F | AGCTCTGCTTGTTACAAAGCC |
|  | R | CAGGTCTTCCAATCTGGATGC |
| PDGFRA | F | AGAGTTACACGTTTGAGCTGTC |
|  | R | GTCCCTCCACGGTACTCCT |
| OXR1 | F | GCAAACTCTGGAAAACTCACTCT |
|  | R | CATCGTAGCACTTGCTTGCG |
| ARL6IP5 | F | GGCAGCGCACAATAAAGACAT |
|  | R | ACACCCCCGAACATGGATATG |
| BNIP3 | F | TCCTGGGTAGAACTGCACTTC |
|  | R | GCTGGGCATCCAACAGTATTT |
| MAPK9 | F | CAGACTGTACCCTCAAGATCCT |
|  | R | CAGACTGTACCCTCAAGATCCT |
| PNPT1 | F | AATCGGGCACTCAGCTATTTG |
|  | R | CAGGTCTACAGTCACCGCTC |
| GAPDH | F | TGGATTTGGACGCATTGGTC |
|  | R | TTTGCACTGGTACGTGTTGAT |
